# New Updates of the Imaging Role in Diagnosis, Staging, and Response Treatment of Malignant Pleural Mesothelioma

**DOI:** 10.3390/cancers13174377

**Published:** 2021-08-30

**Authors:** Chiara Romei, Salvatore Claudio Fanni, Federica Volpi, Alessio Milazzo, Caterina Aida D’Amore, Leonardo Colligiani, Emanuele Neri, Annalisa De Liperi, Giulia Maria Stella, Chandra Bortolotto

**Affiliations:** 1 2nd Radiology Unit, Radiology Department, Pisa University Hospital, 56124 Pisa, Italy; a.deliperi@ao-pisa.toscana.it; 2 Department of Translational Research, Academic Radiology, University of Pisa, 56124 Pisa, Italy; f.volpi2@studenti.unipi.it (F.V.); a.milazzo4@studenti.unipi.it (A.M.); c.damore@studenti.unipi.it (C.A.D.); l.colligiani@studenti.unipi.it (L.C.); emanuele.neri@unipi.it (E.N.); 3 Unit of Respiratory Diseases, Department of Medical Sciences and Infective Diseases, IRCCS Policlinico San Matteo Foundation, University of Pavia Medical School, 27100 Pavia, Italy; g.stella@smatteo.pv.it; 4 Unit of Radiology, Department of Intensive Medicine, IRCCS Policlinico San Matteo Foundation, University of Pavia Medical School, 27100 Pavia, Italy; chandra.bortolotto@unipv.it

**Keywords:** malignant pleural mesothelioma, magnetic resonance, computer-based methods

## Abstract

**Simple Summary:**

Computed tomography plays a pivotal role in malignant pleural mesothelioma imaging management, ranging from diagnosis, differential diagnosis and staging to assessment of therapy response. Indeed, CT still presents some intrinsic limitations such as a poor contrast resolution between tumor and contiguous soft tissues, resulting in a challenging assessment of locoregional staging. Moreover, the current response evaluation criteria are based on unidimensional criteria, while malignant pleural mesothelioma has a complex tridimensional pattern of growth. To overcome these limits, the recent efforts in literature focused on computer-based methods, such as radiomics or automated segmentation, and magnetic resonance imaging. This review aims to describe their potential role in diagnosis, staging and assessment of therapy response in malignant pleural mesothelioma.

**Abstract:**

Malignant pleural mesothelioma is a rare neoplasm with poor prognosis. CT is the first imaging technique used for diagnosis, staging, and assessment of therapy response. Although, CT has intrinsic limitations due to low soft tissue contrast and the current staging system as well as criteria for evaluating response, it does not consider the complex growth pattern of this tumor. Computer-based methods have proven their potentiality in diagnosis, staging, prognosis, and assessment of therapy response; moreover, computer-based methods can make feasible tasks like segmentation that would otherwise be impracticable. MRI, thanks to its high soft tissue contrast evaluation of contrast enhancement and through diffusion-weighted-images, could replace CT in many clinical settings.

## 1. Introduction

Malignant pleural mesothelioma (MPM) is a rare neoplasm that originates from mesothelial cells of pleural, peritoneal, or pericardial tissues [1]. Asbestos exposure is an important risk factor for MPM, although it has been proven that it can be related to mutations in tumor suppressor genes (e.g., *BAP1*), ionizing radiation [2], and environmental exposure [3,4]. MPM usually presents itself with a latency of 30–50 years [2], so this disease is still a global health concern with an increasing incidence [5].

Imaging plays a key role in MPM management because it can provide early detection of pleural disease, differentiation between the benign and malignant process, staging, and evaluation of response to treatments. Among the imaging techniques, chest X-ray examination has a limited role as it is performed in patients with signs and symptoms of pleural pathologies and cannot help in distinguishing benign from malignant process [6].

Fluorine 18 fluorodeoxyglucose (FDG) positron emission tomography (PET) is currently used for MPM staging, although it is not tumor specific and a high uptake of FDG may be seen in benign process, inflammatory disease, or in talc pleurodesis [7]. In addition, tumors with low metabolic activity such as early stage epithelioid MPM may show low FDG uptake in PET/CT examination [8].

**Figure 1:**
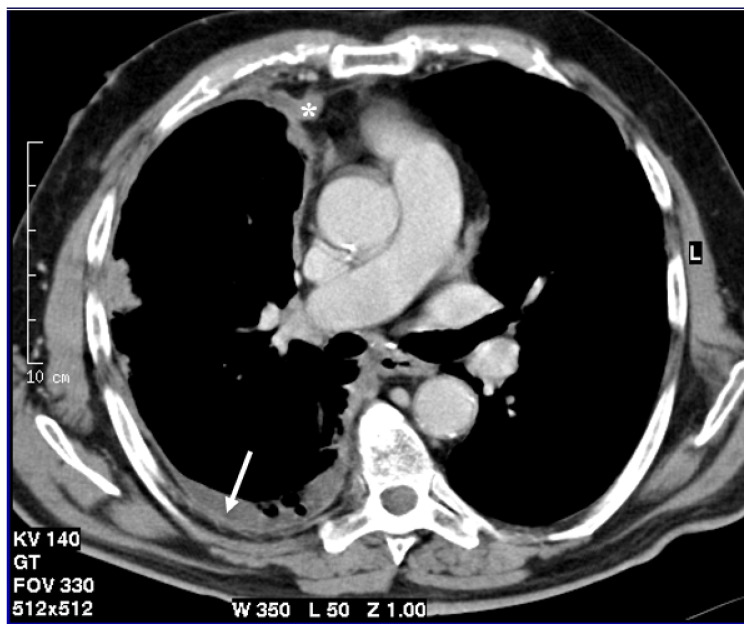
Axial CT scan after contrast administration reconstructed with smooth kernel with mediastinal windows demonstrates circumferential pleural nodular thickening with mediastinal pleura involvement (asterisk) and pleural effusion (arrow).

Computed tomography (CT) with or without an intravenous contrast agent is currently used as the first imaging modality for pleural lesion diagnosis, staging, and monitoring after therapies, as a result of its low cost and its wide distribution [7,9,10]. CT can detect pleural thickening, suggest the presence of the disease, and support the correct execution of biopsies [5]. CT scans without an intravenous contrast agent should be used as screening in risk populations to demonstrate pleural thickening. Once the pleural thickening was evidenced, a contrast agent is necessary to assess specific CT features highly suggestive of MPM as pleural thickening >10 mm, interlobar fissure thickening, mediastinal pleural involvement, and circumferential pleural thickening (Figure 1) [6]. CT is pivotal for clinical staging and subsequentially to patient management [5]. However, CT still has some limits about loco-regional staging and lymph-node metastases evaluation and it is affected by a considerable interobserver variability [8,9]. In particular, CT has a low soft tissue contrast, and this may determine an inaccurate local extent or an imprecise pleural and adjacent involvement; moreover, MPM has an irregular, rind-like, and diffuse pattern of growth, which makes most measurements in single or bidimensional lesions unreliable and not always replicable [11,12,13].

Given the three-dimensional shape of MPM, a volumetric approach seems to be the logical evolution of uni-dimensional criteria. Pass et al. in 1998, demonstrated that the MPM volume measured in a three-dimensional CT performed before surgery correlated with overall survival, but required the manual segmentation of the disease, an extremely time-consuming labor [14]. Since the 90s, a considerable number of other studies have been conducted in order to allow a semi-automatic or automatic segmentation and computing of the tumor volume, thanks also to technological improvements and to the rise in more efficient computer-based methods [11,13]. In addition, computer-based methods have been implemented with many other purposes, from diagnostic tasks with computer-aided detection algorithm to differential diagnosis with radiomics features analysis for the purpose of compensating the intrinsic and above-mentioned limitations of CT.

Chest magnetic resonance imaging (MRI) may be the solution to overcome some limits of CT, as in recent years, MRI has been demonstrated to have a higher spatial resolution [15] and a better soft tissue contrast than CT, with an increase in sensitivity in depicting diaphragm and chest-wall invasion, but not in identifying lymph node metastases and visceral pleural invasion [5,16]. Through MRI, it is also possible to characterize complex pleural effusion such as hemorrhage [17] and although it is not as fast as CT and requires a greater patient compliance, MRI does not have the problem of radiation dose.

The primary aim of this systematic literature review was to summarize the state-of-the-art of computer-based methods and the application of MRI in MPM diagnosis, staging, and response to treatment. The most relevant articles published in the past three decades were reviewed to describe all the investigated applications of chest MRI and computer-based methods in MPM. The review focuses on the potential role of these techniques on MPM and highlights the open issues that future research studies will have to address.

## 2. Materials and Methods

### 2.1. Methods

A systematic literature review was performed to identify all relevant data on radiological assessment of MPM, particularly the role of MRI and quantitative CT analysis on diagnosis and follow-up after surgical and/or medical treatment.

#### 2.1.1. Search Strategies

- Search Sources

The examined databases were Scopus, Web of Science, Cochrane, Google Scholar, and PubMed. The last search was run on February 10, 2021. We have primarily conducted separated research for CT, MRI, radiomics, deep learning and texture analysis. All selected articles were systematically evaluated using the inclusion and exclusion criteria.

For the Google Scholar database, due to the excessive amount of data obtained, only the first 200 results for each search were considered, because further results rapidly lost relevance.

- Search terms

To obtain the highest search sensitivity, the keywords used to identify relevant articles were: malignant pleural mesothelioma OR MPM AND MRI AND magnetic resonance imaging; malignant pleural mesothelioma OR MPM AND MRI AND magnetic resonance imaging AND contrast enhanced magnetic resonance; malignant pleural mesothelioma OR MPM AND CT AND computed tomography; malignant pleural mesothelioma OR MPM AND CT AND computed tomography AND contrast enhanced computed tomography AND radiomics AND deep learning AND texture analysis.

The terms were chosen to include all relevant non-invasive diagnostic approaches to MPM including non-routine ones (e.g., radiomics, deep learning and texture analysis).

#### 2.1.2. Study Eligibility Criteria

In this review, we focused on diagnostic and prognostic aspects of malignant pleural mesothelioma from a radiological point of view, evaluating the strengths and weaknesses of computed tomography, magnetic resonance, and machine learning approaches for both diagnosis and follow-up.

Filters were applied in order to include only articles published in English and described as original research, considering articles published up to 10 February 2021. Any restrictions on the country of publication, comparator, and outcomes were not applied.

#### 2.1.3. Study Selection

Two authors, AM and FV, independently screened the titles of the identified studies. AM and another author (CR) independently screened the titles and the abstracts of the studies that passed the *title* screening; then, they read the full text of studies that passed the *title and abstract* screening. Any disagreement was analyzed and overcome by discussion and reaching a mutual agreement.

#### 2.1.4. Data Synthesis

After collecting the studies and data extraction from the selected articles, we used a narrative approach. In particular, we analyzed different non-invasive types of investigation method for MPM separately and described how their importance and their role have changed thanks to technological evolution, considering the strengths and weaknesses of each method.

### 2.2. Results

#### Search Results

**Figure 2:**
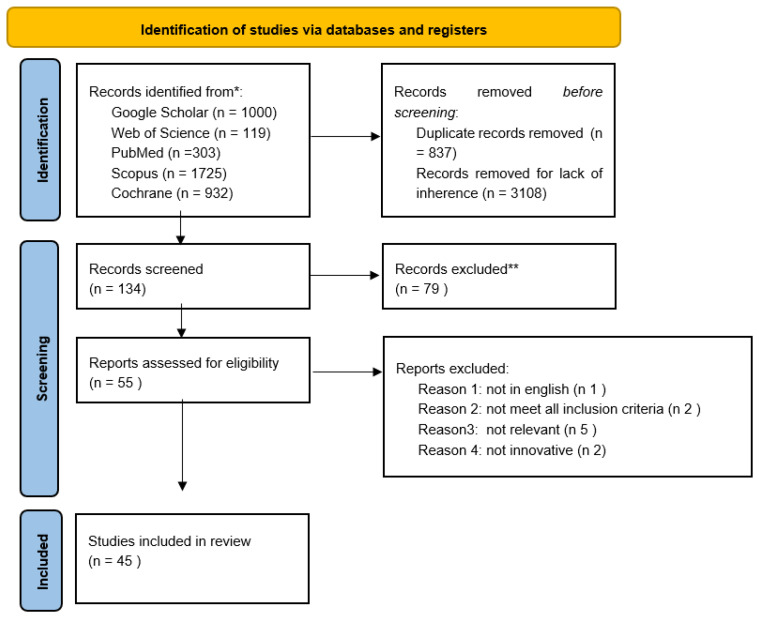
Flow chart of all the harvested papers.

We obtained 4079 studies through searches conducted in the aforementioned databases. Then, we removed 3108 because they were not related to the topic and 837 duplicate records. We screened the titles and abstracts of the remaining 134 studies. After this process, 79 articles were excluded and 55 were assessed for eligibility. After the reading of full text studies, 10 articles were excluded because they did not meet all the eligibility criteria. Overall, 45 studies were included in this review. Figure 2 shows the flowchart for all harvested papers. The studies were then separated into two categories: computer-based methods (Table 1) and magnetic resonance imaging (Table 2).

## 3. Results

### 3.1. Role of Computer-Based Methods in MPM

#### 3.1.1. Introduction

Despite significant progress in MPM management, many of the critical issues above-mentioned, persist. Computer-based methods could represent the solution to overcome some of these issues. There are many computer-based methods based on different software, approach, and aims. Semi-automatic or automatic computer-based methods have been implemented to segment the MPM from surrounding structures, a result that is difficult to obtain without computer assistance due to time limitations and interobserver variability. Moreover, in the last few years, many algorithms have been developed to compute the tumor volume and other quantitative features as well as to extract statistical or radiomics features. Radiomics has been defined as a quantitative approach to medical imaging and its purpose is to extract, quantify, and analyze specific features (shape, intensity and texture) of tumor images [20]. Texture analysis was developed to classify regions through statistical measures of the spatial distribution of gray levels. Recently, an artificial neural network in which higher-level features are extracted from images through multiple interconnected layers to create deep learning (DL) models has been developed.

The specific tasks of computer-based methods can be summarized in three categories: diagnosis, staging, prognosis and therapy response.

#### 3.1.2. Diagnosis

To date, detection of early stage MPM on CT is still extremely difficult due to the intrinsic limitations of CT and the particular growth pattern of the MPM. For this reason, Chaisaowong et al. in 2007 implemented a convexity model used together with a Hounsfield unit (HU) threshold to automatically detect and quantitatively assess pleural thickening on axial chest CT images. To detect pleural thickening, two algorithms were subsequently developed to carry out the segmentation of pleural contours and to perform automatic detection. Detection was based on morphology, as pleural thickenings appear as concave irregularities on the outer pleural surface, density, and thickness excluding all cases with negative Hounsfield values and extension less than three slices. Assessment of characteristic properties (i.e., maximal width and volume of the thickening) was based on a thin plate spline interpolation. Unfortunately, the algorithms were tested only on a limited dataset (only 14 CT from three patients) and neither the detection nor the quantification model performance were validated against experienced radiologist performance [18].

Once the pleural thickening was identified, radiologists use qualitative visual assessment to distinguish between benign and malignant disease. A clear differentiation is not always feasible, in fact, both benign and malignant disease may occur with pleural thickening or effusion [53]. Radiomics could be a potential tool to assist in the interpretation of these complex findings, providing objective and reproducible quantitative information. In 2017, Pena et al. analyzed radiomics features extracted from both CT and MR pleural thickenings to differentiate between the benign and malignant nature, using histopathologic disease as a gold standard [19]. After normalization of the images and manual segmentation of pleural lesions on CT and MR, three textural and three shape features were extracted. Then, combinations of features were inputted as predictors in logistic regression models to compute the ROC curves. The diagnostic accuracy of the model and of both thoracic and abdominal radiologists were then compared. The AUC_CT_ and AUC_MR_ achieved by the best combinations of shape and texture features were 0.92 and 0.87, respectively. The best radiomics model did not outperform the visual assessment of thoracic radiologists on CT and MR, while a better performance of radiomics model was demonstrated compared to abdominal radiologists on both modalities. An important limit of this study is the manual segmentation performed by a single observer that introduce a subjective bias. To underline this limit, in 2018, Pavic et al. demonstrated the importance of inter-observer delineation variability on radiomics analysis, particularly for MPM compared to other tumors. To evaluate the inter-observer segmentation variability, Dice Sørensen coefficient (DSC) was calculated over all possible pairs of the three experienced radiation oncologists. Median DSC result was very low for MPM (0.26), with very low stability for radiomics features (36%) [20].

#### 3.1.3. Staging

Staging is a crucial process to estimate the prognosis, evaluate treatment options, and stratify patients for clinical trials. In 2004, Armato III and colleagues compared manual measurements of tumor thickness with six semi-automatic computerized measurement algorithms, achieving a good agreement (r ≥ 0.95). Slices were selected by a radiologist who identified a starting point at each measurement site, then the algorithm identified an endpoint to delineate a line-segment whose length represented the tumor thickness. Computer-based measurements were highly correlated with the mean of observer measurements (r ≥ 0.93) [26]. In 2005, the same author implemented an interactive interface to evaluate how many times radiologists and oncologists modified the measurement performed by the algorithm, with an encouraging 75% of measurements accepted without modification [27]. These studies by Armato III demonstrated an interesting performance, however, still did not tackle the intrinsic limitation of using a uni-dimensional measurement to describe the bulk of a tumor with an extremely complex growth pattern.

The natural evolution of computer-based uni-dimensional measurement has been the semi-automatic or automatic volumetric approach. Indeed, in 2011, Sensakovic et al. implemented a more complex algorithm to segment the lung parenchyma and non-linear diffusion and k-means classifier to identify MPM findings in the pleural space. This algorithm only required minimal initialization by the user. The performance was then compared to manual segmentation of three observers in a small testing set (31 CT) using the Jaccard similarity coefficient (J). For each of the 31 MPM CT scans, the three observers independently segmented five axial scans. The average J between the computer-based and manual segmentation was 0.506, 0.407, and 0.493 for each observer. Despite there being no significant differences between manual and computer-based segmentation, the algorithm is not valid for a wider clinical application due to some segmentation errors. Most of these errors occur in the lung bases and intercostal spaces and are related to concomitant pleural effusion or atelectasis and to partial volume artifact [28].

In 2017, Chen et al. implemented a semi-automatic random walk-based segmentation method able to segment MPM tumor and to incorporate end-users’ input. The radiologist has more control of the segmentation due to user-defined values, placed in regions known to be tumor, which are the starting point of the segmentation. To validate the segmentation, the algorithm performance was compared to manual segmentation. The median time-spent for the computer-aided procedure was 23.1 min, while for manual segmentation, it was 68.1 min. The DSC was 0.825 and represents a good result in MPM segmentation [29].

The approaches described above, linear and volumetric, were then combined in 2017 by Brahim et al. in two different papers. The proposed methods were developed in three steps: supervised delineation of the thoracic cavity, automatic segmentation of tumor through a statistical texture analysis approach, and finally pleural thickening estimation. The algorithms achieved an interesting J of 0.72 and 0.73 compared with manual segmentation. The differences between the two segmentations were mostly due to algorithm over-segmentation around the mediastinal spaces and under-segmentation in the tumor region [30,37].

For accurate image content analysis and therefore overcome the misclassification problem of the tumor region, deep neural networks may represent a solution. CNNs are a specific type of neural network used in deep learning consisting of multiple layers of convolutional filters that can be trained to recognize image features based-on these features to classify image regions. In 2018, Gudmundsson et al. separately trained two deep CNNs for the left and right lungs to detect pleural thickenings. To train the two CNNs, 4259 and 6192 segmented axial CT sections of the right and left lungs were used. Then, two distinct sets of 131 axial CT sections were used as test-sets to evaluate the performance of the algorithm compared to the manual segmentation performed by eight different observers. As result, the DSC ranged from 0.662 to 0.800, showing more disagreement on test-set 2 due to the greater number of CT-scans with pleural effusions in this set. Misclassification was observed particularly when large pleural effusion was present and misclassified as tumor by the CNNs, leading to a relative difference ≥29.4% between the CNNs and the observers [31].

To overcome this issue, the same authors in 2020 trained a two-dimensional U-net with a dataset of 5230 axial CT sections. A test-set of 94 CT sections with both tumor and pleural effusions was used to validate the network. Despite the extremely complex task, the algorithm achieved a median DSC of 0.690 when compared to manual segmentation, outperforming the past model. This result was achieved thanks to transfer learning, which is a pre-training of the layers on large natural image datasets [32]. Again, the study demonstrated a greater disagreement in manual segmentation between the observers when pleural effusions was shown in the CT scans, raising questions about which gold standard is appropriate to evaluate the performance of these automatic methods.

These intrinsic limitations of manual segmentation can be overcome by using the gross tumor specimen volume as the gold standard. Armato et al. in 2015 compared the CT tumor volume after manual segmentation to the gross tumor specimen volume, resulting in a modest correlation coefficient of 0.66 that further decreased at 0.18 considering only a lower volume of disease [33]. This modest result showed not only the limits of manual segmentation, but also the weakness of using gross tumor volume as a reference standard for MPM segmentation because pathologic volume also includes non-tumor tissue.

The first study that investigated a quantitative staging was made by Gill et al. in 2018, who proposed alternative clinical staging models implementing two quantitative parameters: tumor volume assessed from CT scans (VolCT) and maximal fissural thickness (Fmax). Pre-operative CT scans of 472 patients who underwent macroscopically complete surgical resection of MPM were analyzed by a radiologist both qualitatively, using the American Joint Committee on Cancer (AJCC) Staging Manual, and quantitatively, assessing tumor volume by semiautomatic segmentation (VolCT) and measuring the maximum thickness of interlobular fissural thickenings (Fmax). Pathological staging for each patient was also evaluated and used as the gold standard. One third of the patients in the study cohort was assigned to a training set to generate staging models inclusive of the quantitative parameters; the performance of these models was evaluated in a test set including the remaining 2/3 of the patient cohort. Aiming to create quantitative models that could be a surrogate to clinical staging, VolCT and Fmax values were transformed into categorical variables; considering values derived by hazard ratio and overall survival analysis in the test set, the process resulted in a four-level bivariate model with four stages based on VolCT, with upstaging by one level if a Fmax threshold was surpassed. The performance of the staging models as prognostic classifiers was assessed with the Harrel’s C index; the baseline clinical AJCC showed a modest performance (c-index = 0.562), while the quantitative models had a superior discriminative performance statistically significant both when using VolCT alone (c-index = 0.629, *p* = 0.004) and the bivariate four level model (c-index = 0.638, *p* = 0.001) [35].

The first multicenter volumetric CT study was performed by Gill et al. in 2016. Two radiologists independently performed a semi-automatic volumetric assessment of MPM through manual editing after an initial automated tumor segmentation based on HU value. The correlation between the measurements of the two radiologists was good (Spearman Corr. = 0.822), an accurate analysis of discordant cases showed that they were attributed mostly to perception errors, data entry errors, and user errors in tool knowledge [34].

The above-mentioned papers measure quantitative parameters that are directly related to the tumor, but other parameters must be mentioned such as the thoracic cage volume (TCV). Burt et al. in 2020 investigated the incidence of preoperative predictors of diffuse chest wall invasion (DCWI), the most common factor precluding macroscopic complete resection (MCR) of MPM. DCWI is generally assessed by the surgeon at the time of resection; a preoperative identification of DCWI is necessary to avoid worthless thoracotomy, and to save time in order to start non-surgical therapy patients with MPM as early as possible. Several potential predictive factors were evaluated in two cohorts of patients, one with MCR (n = 143) and one with DCWI (n = 27). TCV was computed on pre-operative CT scans applying 3D volume analysis in imaging software. In univariable regression analysis, a reduction in TCV ipsilateral to the MPM compared to the contralateral demonstrated an interesting and strong association with DCWI (*p* = 0.009); additionally, the entity of the ipsilateral TCV reduction was demonstrated to be significantly higher in patients with DCWI, showing an AUC of 0.67% and setting as cutoff a percentage change value in TCV of −5% [36].

#### 3.1.4. Prognosis and Therapy Response

The research of prognostic factors and scores to carefully select candidates for curative purposes and potentially toxic multimodal treatment is still ongoing. In fact, current staging systems do not effectively stratify prognosis [54]. The first potential prognostic factor, as can be easily conceived on the basis of the reports above-mentioned, is the tumor volume. In 2012, Gill et al. investigated the prognostic value of pre-operative tumor volume CT together with other clinical factors in a cohort of 88 patients with histologic features of epithelial MPM treated with adjuvant chemo/radiotherapy and extrapleural pneumectomy. The tumor volume was identified and segmented with 3D volume software, while extrapleural sites or discontinuous regions of involvement were identified and manually added by a thoracic radiologist. Pre-operative tumor volume and hemoglobin concentration had strong results in multivariate analysis and was independently associated with survival after extrapleural pneumectomy. A threshold of 500 cm^3^ for pre-operative tumor volume showed a hazard ratio of 2.02 and resulted in the strongest prognostic factor; moreover, it was independent of clinical stage [21]. However, these results need to be generalized to nonepithelial disease and to other types of cytoreductive procedures.

Additionally, radiomics features could be useful as prognostic factors. In 2020, Pavic et al. extracted 1404 CT and 1410 FDG PET-CT features from pre-operative exams of 72 MPM patients treated with curatively intended extrapleural pneumectomy or pleurectomy/decortication. Primary tumors were manually segmented by four radiation oncologists and subsequently shape, intensity, texture, and wavelet features were extracted. Only stable features independent of inter-observer segmentation variability were considered and non-redundant features were included in the multivariate Cox regression analysis. As a result, only the final FDG-PET radiomics model was predictive for progression free survival (PFS) (concordance-index = 0.66), while the CT radiomics model could not be successfully validated [22].

Accurate and reproducible assessment of disease response to therapy is crucial to evaluate the efficacy of such therapy in both clinical trials and clinical practice. Compared to the traditional methods, a more accurate and reproducible approach could be the comparison of overall tumor volume before and after treatment. Manual segmentation of the entire tumor volume would be extremely time-consuming and presents some interobserver variability. Driven by the aim of overcoming the mRECIST and entire volume manual segmentation issues, Labby et al. in 2013 performed manual area measurements of the MPM findings. Baseline and first follow-up CT scans were obtained for 31 patients. In a similar manner to the mRECIST, three sections with significant MPM findings were selected by a single experienced radiologist and then five radiologists proceeded to independently contour the tumor for each baseline and follow-up CT scans. For each lesion contoured, the area was calculated using Green’s theorem; the areas for each CT scan were then summed to produce a “pseudovolume”, that is, the volume of disease on a small subset of slices of CT. However, the results showed a high inter-observer variability due to different approaches in contouring lesions or different perceptions of tumor. This study, according to previous ones, demonstrated that manual segmentation, although limited to only three slices, has inter-observer variability that prevents meaningful and reliable response assessment. Moreover, the time required for each segmentation was to the order of 20 min per scan, still too long time for implementation in clinical practice [12].

Computer-aided measurement techniques could improve the time needed to segment tumor volume and the reproducibility of the process. In 2010, Liu and colleagues used a semiautomatic computer-aided method to measure tumor volumes in 30 MPM patients from two clinical trials, before and after chemotherapy. mRECIST measurements were also available. The computer method segmented the tumor from adjacent non pathological tissues (i.e., lungs, chest wall, heart etc.); two radiologists then corrected each scan independently to assess interobserver reliability. It was found that a change in tumor volume was significantly associated with overall survival (HR: 1.94, *p* = 0.04); in contrast, mRECIST could not predict a change in overall survival (HR: 1.06, *p* = 0.25). The volumetric measurement showed a significantly higher predictive ability than that of mRECIST (C-index = 0.74 vs. C-index = 0.5, *p* = 0.05). The patients were divided into two groups based on the increase or decrease in tumor volume, and a significant difference was found in median survival between the two groups (11.5 months vs. 18.1 months, *p* = 0.03). This method showed good reproducibility: the concordance of correlation coefficients between the two radiologists for both baseline (CCC = 0.993) and follow-up (CCC = 0.991) measurements were good [24].

Similarly, in 2013, Labby and colleagues created a model in order to analyze tumor volumes continuously during chemotherapy, in conjunction with clinical covariates, to predict survival. An average of four scans per patient were obtained from 81 patients at baseline and during follow up after chemotherapy. The tumor volumes were segmented using a semiautomated method with manual editing requiring 10–20 min per case. The disease volume, through continuous and time varying measurements, was modeled as the specific growth rate (SGR) from baseline according to a logarithmic equation; the SGR was found to be predictive for survival in univariate proportional hazard models [23].

In 2011, Frauenfelder et al. also evaluated the accuracy of volumetric measurement before and after therapy compared to the mRECIST criteria. In this study, three readers independently analyzed CT scans from 30 patients before and after therapy, presenting their measurements according to mRECIST criteria and with a volumetric approach. For the latter, they used dedicated software (Myrian, Intrasense, Paris, France), which was previously developed for liver segmentation, to measure tumor volume following three steps: normal lung was segmented semi-automatically, pleural effusion and atelectatic lung were excluded manually, and the outer edge of the pleura was segmented semi-automatically. The three readers were tasked to manually segment only one every 4–5 slices, leaving the rest of the work to an interpolation algorithm, thus drastically reducing the time needed to perform the segmentation to 10–15 min per case (but still higher than the 3 min required to apply mRECIST measurements). When comparing the classification of tumor response according to mRECIST criteria, it was found that there was a mismatch between the readers in 16 cases out of 30, suggesting a moderate interrater agreement (kappa = 0.33). On the other hand, there was no mismatch between readers with the volumetric approach, with a k value of 0.89, indicating an excellent inter-rater agreement [25].

### 3.2. Role of MRI in MPM

#### 3.2.1. Introduction

The role of magnetic resonance imaging (MRI) in the assessment of pleural diseases has grown in the past years and is already considered the imaging of choice in the evaluation of superior sulcus carcinoma [40]. MRI advantages include the absence of ionizing radiation, the high soft tissue contrast, and the intrinsic flow sensitivity [41]. Some of the greatest limitations to MRI use are its high costs and the poor availability of MRI scans. Therefore, the advantage of CT guided biopsy compared to MRI utility has been proposed as an alternative to improve pleural disease differential diagnosis [6]. Because MRI evaluation is not related to radiation exposure, it could be a suitable tool for repeatedly screening programs and in monitoring patients treated with chemotherapy [38].

#### 3.2.2. Diagnosis

**Figure 3:**
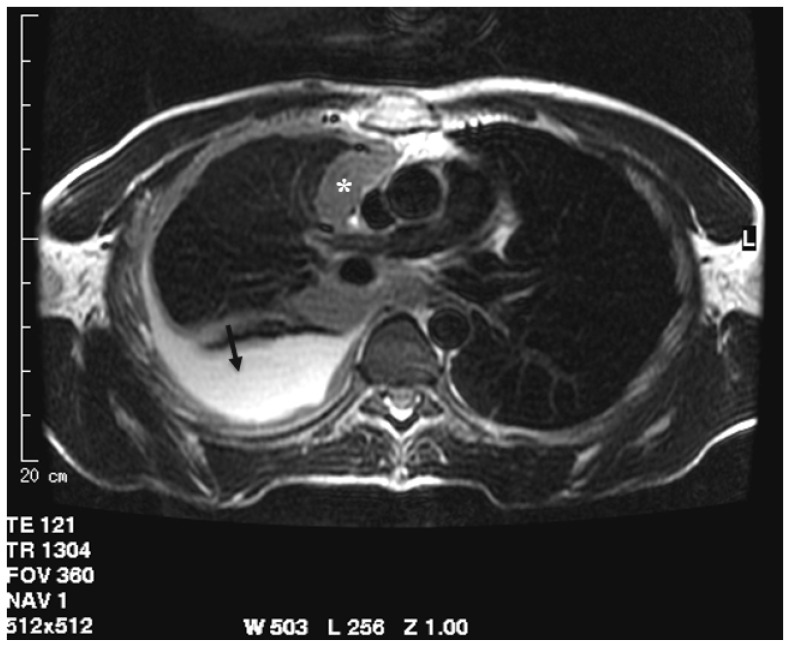
T2-weighted image acquired in the axial plane clearly demonstrates circumferential pleural thickening with mediastinal pleura involvement (asterisk) and pleural effusion (arrow).

One of the first studies regarding the role of MRI in assessing pleural disease evaluated the morphological appearance of benign and malignant lesions and their differential diagnosis. In 1999, Boraschi et al. analyzed the appearance of MRI in asbestos-related benign and malignant pleural disease. Thirty patients underwent MRI examination due to the previous detection of suspected pleural tumor on CT. The differential diagnosis was based on morphological features like thickness of pleural lesion, regularity of internal and external margins, mediastinal pleural involvement, circumferential pleural thickening or “pleural -rind”, pleurisy, diffusion to other structures, and signal intensity in T1 and T2 weighted images [40]. The assessment of a lesion with a thickness lower than 1 cm, regular margin, hypo, or isointense signal intensity on T2-weighted sequences (compared to that of muscle) and homogenous contrast enhancement were defined as signs of benignity, whereas malignant lesions were characterized by thicknesses greater than 1 cm, irregular margins, mediastinal pleural involvement, pleural “rind”, pleurisy, and extension to other thoracic structures. The signal intensity of the malignant process was low-to-intermediate in T1-weighted images and inhomogeneously hyperintense both in the proton density and T2-weighted images, with significant enhancement in T1 weighted sequences after intravenous paramagnetic contrast administration. A sensitivity of 100% and a specificity of 95% of MRI in the detection of pleural malignancies was highlighted. The extension of the tumor was better evaluated in MRI than CT, especially in the coronal plane, assessing the apical, diaphragmatic, infradiaphragmatic involvement and the relation with mediastinal structures (Figure 3) [40].

MRI superiority in morphological evaluation of pleural lesions and in assessing locoregional extension was also demonstrated in two other studies: one by Knuutila A. et al. in 1998 [52] and the second by Falaschi et al. in 1996 [51]. In the latter work, it was reported that signal hyperintensity in proton-density and T2-weighted images in pleural malignancies (like mesothelioma, metastasis, or non-Hodgkin’s lymphoma) demonstrated a 100% sensitivity and a 87% specificity [51]. Mesothelioma and secondary pleural involvement such as lymphoma or metastasis represent 10% and 90% of pleural malignancies, respectively [7,9].

In 2000, Hierholzer et al. analyzed the role of CT and MRI in differential diagnosis of pleural disease in 42 patients previously selected on the basis of the presence of pleural thickening on CT. Again, the major CT features related to malignant nature were mediastinal pleura involvement, diffuse and circumferential pleural thickening, evidence of nodularity or irregularity of pleural contour, and finally infiltration of diaphragm and/or chest wall [6].

The detection of pleural calcification on CT was suggestive for benign disease even if some benign diseases may mimic a neoplastic process as reactive pleurisy, although it usually spares mediastinal pleura and tuberculous empyema [40]. In the end, MRI was more sensitive in the assessment of chest wall and diaphragm involvement with MRI morphological and signal intensity features, which showed a sensitivity of 100% and specificity of 93% in malignant pleural process detection [6].

In the following years, the 3-Tesla-high-field magnetic resonance scan was improved, and in 2010, Podobnik et al. evaluated fifteen patients with asbestos-related pleural disease. In this study, the proposed MR protocol was: T2-weighted cardiac-gated breath-hold turbo spin echo (TSE) sequences in three planes, a T1-weighted cardiac-gated breath-hold TSE black blood sequence, and a T2 weighted-spectral pulse inversion recovery (SPIR) sequence with fat saturation signal. The signal intensity differences between the benign and malignant process were the same reported in the previous studies above-mentioned and no significant differences between MRI and CT evaluation were highlighted. However, a small number of patients was analyzed [38].

In the last few years, despite the morphological features detectable on MRI, more importance to quantitative analysis has been given thanks to the introduction of diffusion weighted imaging (DWI) and dynamic contrast-enhanced sequences (DCE). DW imaging contrast sets its basis on the random Brownian motion of water protons in a tissue [55]. From this noninvasive technique, it is possible to obtain quantitative information of water molecule mobility using apparent diffusion coefficient (ADC) maps [7]. Briefly, ADC values are generally measured automatically by software and are then shown as a parametric map that displays the degree of diffusion of water molecules in different tissues [56]. Both DWI and ADC could help in differentiating benign from malignant lesions and in distinguishing false positive PET/CT pleural disease as inflammation and talc pleurodesis [7].

In 2012, Coolen et al. evaluated the role of DWI and DCE sequences in pleural lesion differential diagnosis. The optimal ADC cut-off value highlighted was 1.52 × 10^−3^ mm^2^/s. However, ADC may provide false-negative results due to the presence of intra-tumoral necrosis or inflammation. When the ADC was between 1.52 and 2.00 × 10^−3^ mm^2^/s, perfusion parameters were used to assess the malignant nature of the lesion, then improving the accuracy with ADC. The addition of DCE to DWI-ADC analysis improved the sensitivity from 71.4% to 92.8%, but the specificity decreased from 92.8% to 94.1% with an overall accuracy of 93.5% [7].

The relevance of DCE sequences was highlighted in another study by Knuuttila et al. in 1998, in which the enhancement of interlobar fissures was described as a feature suggestive of MPM together with the already known morphological features. The superiority of MRI compared to CT in loco-regional staging was also confirmed [39].

In the study by Mehndiratta A. et al. in 2009, the role of DCE was analyzed by radiologists who assessed both the diagnostic significance and tumor vasculature display examining a sequence of two different functional MRI post processed display methods: color coded and grey-scaled images. A higher diagnostic quality as well as a more accurate tumor vasculature in DCE-MRI were demonstrated by color coded rather than grey scale images.

Thus, since even unperceivable changes are better distinguished in color coded display, an extended use of this technique should be considered [44].

In a more recent study by Tsim et al. (2018), MRI early contrast enhancement (ECE) of pleural lesions was analyzed. Sixty patients were included in the study. First, benign and malignant lesions were classified based on morphological evaluation. Then, perfusion analysis was performed by placing from five to 15 ROI (region of interest) on the pleural mass lesion or, where a lesion was not identified, randomly. If ECE was not detected in any of the ROIs and morphological appearance was typical for a benign nature, the lesion was classified as benign. If morphological appearance was typical for malignancies, a diagnosis of MPM was proposed. Otherwise, if morphological presentation was benign but ECE was assessed, malignant nature was indicated. Of the 60 patients, 36 had malignant lesions, five had secondary pleural malignancies, and 31 MPM. Altogether, ECE showed a higher diagnostic sensitivity (83%) and negative predictive value (92%), outperforming subjective morphology evaluation both on CT without perfusion analysis and on MRI (56% and 67%, 68% and 78%, respectively). ECE was characterized by a higher interobserver agreement (k 0.784) compared to CT (k 0.65) and MRI morphology (k 0.593) [8].

In another study by Coolen et al. in 2015, the relevance of visual assessment of “*pleural pointillism*” on high b-value DWI sequences was highlighted as a useful tool in differential diagnosis between benign and malignant pleural diseases. *Pleural pointillism* means the presence of multiple hyperintense spots on high b-value DWI because it is visually reminiscent of the Post-Impressionistic painting technique known as *Pointillism.* The advantage of pleural pointillism is its detection in early stages of MPM. In addition, it could be used to identify the optimal biopsy sampling site together with PET/CT information [41]. Another relevant feature reported in this work was the “*shrinking lung”,* the contraction of the affected hemithorax, although this may also be seen in other pathologies, mostly autoimmune disorders such as systemic sclerosis, systemic lupus erythematosus, and Sjogren syndrome or as consequences of chronic empyema and hemothorax [41].

The role of the DWI sequence in differential diagnosis of pleural disease was confirmed in a retrospective study by Revelli et al. in 2016, in which respiratory triggered DWI sequences were applied in a group of 56 patients with suspect malignant pleural tumor. Respiratory triggered DWI provides a more accurate measurement of ADC values despite a longer acquisition time, which can be reduced using high value parallel imaging. All patients underwent thoracoscopic biopsy and MRI exam: 44 patients had a histological diagnosis of MPM (31 epithelioid, four biphasic, and nine sarcomatoid). The ADC optimal cut-off value was 1.5 × 10^−3^ mm^2^s^−1^ with a sensitivity of 100% and specificity of 91.67%. The correlation between different subtypes and ADC values was analyzed: a higher value of ADC in the epithelioid subtype was highlighted, while in sarcomatoid, lower ADC mean values were linked to the frequent more extensive intratumoral necrosis and cellular edema [9].

A cut-off ADC value of 1.28 × 10^−3^ mm^2^s^−1^ was highlighted in another study by Koc et al. in 2017, in which a group of 62 patients underwent MRI examination and subsequent histological diagnosis showed a lower specificity, sensitivity, and predictive value of ADC compared to those in the previous studies mentioned [42].

DWI utility in differentiating between different MPM histological subtypes was confirmed in a study by Gill R.R. et al. in patients affected by MPM and ADC maps were computed from the DWI sequence. The results showed good correlation between ADC values and the three different histologic subtypes. ADC value of 1.31 × 10^−3^ mm^2^/s was typical for epithelioid MPM, 1.01 × 10^−3^ mm^2^/s for biphasic MPM and 0.9910^−3^ mm^2^/s for sarcomatoid MPM. As a result, the ADC value of the epithelioid subtype was compared to the sarcomatoid subtype and was found significantly higher *p* < 0.05). No significant differences between the ADC values of biphasic and sarcomatoid MPM were revealed [43].

In a recent study by Usuda et al. published in 2019, the role of DWI sequences in differentiating between pleural dissemination of lung cancer, empyema, pleural effusion, and mesothelioma was analyzed. DWI was confirmed as a valid tool in differentiating between the malignant and benign process and was even able to differentiate between MPM and pleural dissemination of lung cancer [50].

#### 3.2.3. Staging

PET/CT proved to be the most precise technique in MPM staging with a higher accuracy compared to other imaging modalities such as CT and MRI, thanks to the precise evaluation of lymphatic nodes and the early detection of initial MPM. However, PET/CT showed some limitations in the evaluation of thoracic wall invasion because of the limited spatial resolution. As reported in a study by Plathow et al. in 2008, and as already explained in the mentioned studies regarding MRI morphological evaluation of MPM, MRI is a more suitable tool in assessing locoregional invasion, particularly in diaphragm and infra-diaphragm invasion. T2-weighted HASTE images have been proven to better identify pleural effusion compared to CT. MRI also has a higher contrast resolution, resulting in a better delineation of the desmoplastic reaction and differentiation between tumor tissue and contiguous connective tissue [47].

In a work by Tsim et al. in 2020, volumetric analyses performed on CT and MRI were compared. Interobserver agreement and accuracy were higher for MRI volumetric analysis because of the improved contrast resolution between the tumor and the surrounding soft tissues [13].

In the differentiation of T3 and T4 stage disease, contrast enhanced (CE)-MRI also appeared as a reliable method to provide an accurate evaluation of tumor extension to assess resectability, as demonstrated by Stewart et al. in 2003. It is unlikely to contribute significantly to nodal staging, but it remains a helpful adjuvant in the selection of patients designated for radical surgery [48].

According to the study of Patel et al. in 2017, the best time delay to program CE-MRI and achieve the peak of MPM tumor enhancement is between 150 and 300 s after contrast administration. This specific time range correlates with a better tumor perception when MR images are viewed by radiologists. This result was obtained by comparing the measurable tumor enhancement with a qualitative difference in tumor dimension perceived by radiologists. Thus, an optimal tumor enhancement could improve the clinical interpretation of exams performed for MPM, further increasing the conspicuity of the tumor compared to contiguous soft tissues [57].

Heelan et al., in 1999, according to the International Mesothelioma Interest Group staging system, evaluated patients using both CT and MR imaging. MRI showed a higher sensibility to reveal involvement of chest wall, endothoracic fascia involvement, and diaphragmatic muscle. However, no statistically significant differences were found between CT and MR when MPM staging was performed [16].

Ohno et al., in 2019, demonstrated a higher diagnostic accuracy for TNM stage assessment of whole-body MRI and of combined imaging modalities such as FDG PET/MRI, and FDG PET/CT compared to conventional imaging examination. In particular, whole-body MRI and FDG PET/MRI outperformed conventional imaging for nodal assessment and evaluation of the MPM stage [15].

#### 3.2.4. Assessment of Therapy Response

To estimate tumor response after therapy, mRECIST and volumetric approaches present the most accurate scores. In contrast, CT and conventional RECIST proved to be not as precise. MRI (HASTE, VIBE, T2-TSE sequences) was superior in detecting soft tissue contrast even without intravenous contrast media administration and better delineates the local extent of MPM. In summary, in the study of Plathow et al. in 2008, it was assessed that to evaluate early therapy response, mRecist criteria applied to MRI are recommended, however, in the evaluation of pleural lesion, nowadays, CT remains the gold standard, even for mRecist application, especially in patients with limited physical status and breathing problems because of the shorter acquisition time [46].

Further studies have attempted to prove the role of MRI in the assessment of MPM response to chemotherapy. In 2006, Giesel et al. investigated the potential role of DCE-MRI and the pharmacokinetic parameters of MPM enhancement after contrast administration to assess biological effects in patients undergoing chemotherapy. Indeed, DCE-MRI with the implementation of these new parameters may help to identify microscopic features such as microvascular properties and tumor heterogeneity while monitoring response to chemotherapy. The pharmacokinetic parameters computed to characterize the tumor region were amplitude (Amp), redistribution rate constant (kep), and elimination rate constant (kel). A correlation was found between pharmacokinetic kep values and poor overall response to therapy. MPM has a rapid contrast uptake and washout in relation to an increased neo-angiogenesis and vascular permeability. High tumor expression of pro-angiogenetic factors (VEGF, VEGF type C) in MPM is associated with shorter survival. New therapeutic antivascular-targeted agents like anti-VEGF antibodies require functional imaging, in order to detect the response and facilitate personalized treatment as early as possible, so MRI seems to be a promising tool [45].

In a recent study by Tomsic M.V. et al. (2019) DCE was proposed as a reliable biomarker for the evaluation of chemotherapy response. In this work, two kinetic models for DCE-MRI analysis were tested, Extended Tofts (ET) and adiabatic approximation tissue homogeneity model (AATH) and were compared to mRecist evaluation. Perfusion analysis turned out be more sensitive in detecting early tumor response to therapy [49].

## 4. Conclusions

CT scan is still the primary imaging modality used for MPM evaluation. Despite this, the CT features for MPM detection have a low negative predictive value, even more so in the early stage of disease [6]. Meanwhile, MRI is characterized by better sensitivity and specificity through the combination of morphologic features, and signal intensity information confers a higher sensitivity for the detection of pleural malignancy.

Regarding staging, the critical distinction to perform is differentiation between resectable (T3) and unresectable (T4) tumors. The role of CT to assess the T parameter is limited by a low contrast resolution between the tumor and chest wall, whose involvement defines T4 staging. Indeed, CT scans could underestimate the stage of disease, especially in cases of early invasion [16]. MRI has the advantage of a higher contrast resolution, resulting in better delineation between tumor tissue and contiguous connective tissue [47]. In selected cases, MRI should be used to detect the initial involvement of the diaphragm, lung apex, or thoracic wall.

To assess response to treatment, the morphology and asymmetric growth of the tumor still represent significant challenges for accurate measurement of tumor burden on CT. Computer-based methods have demonstrated their potential role in filling this gap. Semi-automated and, moreover, automatic methods make feasible the extremely difficult and time-consuming task of segmentation, opening to other possibilities such as volume computing or the extraction of radiomics features. These quantitative parameters could be implemented in clinical practice, as has already been demonstrated through their role not only in the assessment of therapy response, but also in diagnosis and staging. Moreover, DWI and DCE MRI sequences increased accuracy, providing elements that may be used for assessing chemotherapy response instead of or in combination with the well-known mRecist criteria when using novel drugs as the anti-VEGF, etc.

The limits of MRI and computer-based methods may be summarized in the paucity of MPM incidence, indeed, the cohort are often limited in number, making the results not generalizable. Most of the mentioned studies included patients with already known pleural lesions: this caused a bias in evaluating the accuracy/sensitivity of various imaging modalities. For studies on computer-based methods, the dataset implemented is often not public and even less the algorithm implemented, making it difficult to reproduce the results or directly compare different methods. The limits to MRI application in pleural lesion evaluation are its high costs, the poor availability of scanners, and a longer acquisition time compared to those of CT. However, MRI should be considered in order to avoid more invasive examination such as CT guided biopsy or thoracoscopy, especially in patients with poor performance status.

In conclusion, MRI and computer-based methods are new techniques with the potential to overcome the issues of CT in MPM imaging management. However, to introduce these new techniques in clinical practice, new prospective studies with a larger cohort of patients will be necessary.

## Figures and Tables

**Table 1:** Summary of studies on computer-based methods included in the review.

Publication	Computer Based Method	Types of Data	N Patients	Problem/Assignment	Validation Method	Accuracy/Results
Chaisaowong K. et al., 2007 [18]	A convexity model with a Hounsfield Unit threshold was implemented to detect pleural thickness	CT	3	To develop an automatic image processing approach to detect and quantitatively assess pleural thickenings		
Pena E. et al., 2017 [19]	Combinations of radiomics features used to generate logistic regression models. 3 texture and 3 shape features	CT and MRI	34	To identify a radiomic approach that may help differentiate benign versus malignant pleural lesions	Visual assessment by thoracic and abdominal radiologists	CT model revealed an AUC of 0.92 ± 0.05 outperforming abdominal radiologists
Pavic M. et al., 2018 [20]	An in-house developed software was implemented to extract features from manually contoured CT scans	CT	11	To investigate the impact of inter-observer variability in manual tumor delineation on the reliability of radiomic features	3 experienced radiation oncologists manually segmented CT scans	Median Dice Sørensen coefficient (DSC) was low (0.26) with a low stability rate of radiomic features (36% of total parameters)
Gill R. R. et al., 2012 [21]	Selective segmentation with the 3D feature of the software, with manual segmentation of extrapleural sites of disease	CT	338	To assess the usefulness of CT-derived tumor volume for stratifying survival after surgery-based multimodality treatment		At multivariate analysis a tumor volume > 500 cm3 showed a HR = 2.02, *p* = 0.0109
Pavic M. et al., 2020 [22]	Extraction of CT and FDG PET features to build a Cox regression model	FDG PET and CT	123	To build a CT and FDG PET radiomics model for the prediction of prediction free survival (PFS) in MPM		Concordance index of 0.66 was obtained for the PET radiomics model, CT radiomics model not successfully validated
Labby Z. E. et al., 2013 [23]	Semiautomated segmentation with semiautomated shape-based interpolation requiring seeding	CT	81	To create a comprehensive model for MPM survival utilizing continuous, time-varying estimates of disease volume from CT imaging in conjunction with clinical covariates		Final multivariate survival model included continuous specific growth rate from baseline (HR = 1.31)
Fan Liu, et al., 2010 [24]	Semiautomated segmentation method combining chest-rib interpolation, gradient vector flow snake and multiple thresholding technique with manual editing of suboptimal results by a thoracic radiologist	CT	30	To calculate the tumor volume and to investigate whether the baseline volume or volume change after chemotherapy predicts patient survival	A second radiologist independently reviewed the computer results	Percentage change of tumor volume from baseline to first follow-up CT was significantly associated with overall survival (HR = 1.94)
Labby Z. E. et al., 2013 [12]	5 observers manually contoured tumor on 3 selected sections, then contours were converted to area measurements using Green’s theorem	CT	31	To evaluate manual area measurements as an alternate response assessment metric, specifically through the study of measurement interobserver variability		The time required to contour tumor for each scan was 20 min; the 95% CI for relative interobserver variability for summed area measurements was [−71%, +240%] for baseline and [−41%, +70%] for FU scan
Frauenfelder T. et al., 2011 [25]	A dedicated software semiautomatic feature with linear interpolation was implemented to segmentate MPM and compute the tumor volume	CT	30	To assess robustness of volumetric measurement of MPM before and after chemotherapy compared to mRECIST criteria	3 readers independently assessed the tumors response	For tumor volume compared to mRECIST were found a high inter-rater reliability (0.99) and inter-observer agreement (general k 0.9)
Armato III S. G. et al., 2004 [26]	6 computerized algorithms (from Minimum-distance algorithm to Normal-to-initial-end-point algorithm) given a specified initial endpoint measured tumor thickness	CT	22	To evaluate the variability of manual MPM thickness measurements in CT scans and to assess the relative performance of six computerized measurement algorithms	5 observers manually measured tumor thickness	Computer based tumor thickness measurements highly correlated with the average of observer measurements (R ≥ 0.93)
Armato III S. G. et al., 2005 [27]	A semiautomated method computes tumor thickness requiring the manual selection of a point in the outer margin of tumor	CT	22	To evaluate the clinical acceptability of semiautomated methods for the measurement of MPM thickness in CT scans	3 radiologists and oncologists independently reviewed measurements	86% of semiautomated measurements were accepted without modification
Sensakovic W.F. et al., 2011 [28]	An automated method based on grey level, texture and shape analysis segmented lung and nonlinear diffusion and a k-means classifier identified MPM in the pleural space	CT	31	To present a computerized method for the three-dimensional segmentation and volumetric analysis of MPM	3 observers independently contoured 5 randomly selected sections for each scan	The median Jaccard index between the computer based and manual segmentation was 0.484
Chen M. et al., 2017 [29]	A random walk-based algorithm was implemented to segment the tumor	CT	15	To assess the performance of a computer-aided semi-automated algorithm for the purpose of segmenting MPM on CT	Manual delineation by a clinical radiologist	A mean DSC of 0.825 was achieved; a Pearson’s correlation coefficient of 0.6392 was established between changes in mRECIST and tumor volume
Brahim W. et al., 2019 [30]	After supervised delineation of thoracic cavity the tumor was automatically extracted through a statistical texture analysis approach	CT	10	To propose a diagnostic aid system capable of segmenting and measuring the pleural thickening caused by MPM	Manual segmentation of a representative database	The algorithm obtained an average Jaccard index of 0.72
Gudmundsson E. et al., 2018 [31]	Two convolutional neural networks (CNN) were trained to segmentate pleural thickenings of left and right hemithorax	CT	130	To automatically segmentate MPM on CT scans using CNNs	Manual segmentation of 8 different observers	Median DSC ranged from 0.662 to 0.800 over the two test sets
Gudmundsson E. et al., 2020 [32]	Two CNNs were trained for segmentation of tumor implementing layers pretrained on ImageNet	CT	203	To automatically segmentate MPM on CT scans using CNNs also in more complex scenarios as of pleural effusion	Manual segmentation on 2 different test sets	Median DSC of 0.69 on the tumor and a fusion test set
Armato III S. G. et al., 2015 [33]	Computation of CT-based tumor volume, after manual segmentation by a radiologist, as a number of pixels	CT	28	This study evaluated the validity of image-based tumor volume against the physical volume of the tumor bulk		A correlation coefficient r-squared value of 0.66 was found
Gill R. R. et al., 2016 [34]	Semiautomated segmentation using HU thresholding with manual editing (exclusion of pleura effusion and chest wall musculature) by 2 thoracic radiologists	CT	129	To assess feasibility and logistics of setting up a quantitative imaging study for clinical staging of MPM	AJCC pathological staging was assessed by the two radiologists on preoperative CT scans	A good overall correlation between computed tumor volume was found (Spearman Corr. = 0.822); tumor volume correlated with pathological T stage (results are reported in a separate manuscript)
Gill R. R. et al., 2018 [35]	Semiautomated segmentation with HU thresholding and manual correction to exclude pleural fluid and normal tissue; a software integrated measurement caliper was used to measure maximal fissural thickness	CT	472	To improve prognostic classification of MPM exploring alternative staging models based on quantitative parameters such as volume assessed from CT scans (VolCT) and maximal fissural thickness (Fmax)	AJCC pathological staging information were obtained from the electronic medical record for each patient	A quantitative model with both VolCT and Fmax was found to be a better prognostic classifier compared to cTNM (c-index = 0.638, *p* = 0.001)
Burt B.M. et al., 2020 [36]	The 3D volume feature of the software was implemented to render the thoracic cage and, after manual removing of undesired objects, to calculate TCV (thoracic cage volume)	CT	170	To determine the incidence and preoperative predictors of diffuse chest wall invasion		In univariable analysis decreased TCV demonstrated the strongest association with diffuse chest wall invasion (*p* = 0.009)
Brahim W. et al., 2017 [37]	A texture analysis method based on statistical approach was implemented to segmentate MPM	CT	10	To present a texture-based segmentation method of the MPM from thoracic CT scans	Tumoral regions were manually contoured and used as ground truth	The average Jaccard index was 0.73

**Table 2:** Summary of magnetic resonance studies included in the review.

Publication	Study Design	Study Population (Period, Location)	N Eligible Patients	N Included Patients	N MPM Patients	Imaging Technique	MRI Sequences
Podobnik J. et al., 2010 [38]		Not reported	15	15	10	3T MR and CT	T2-weighted cardiac-gated breath-hold TSE sequences, and T1-weighted cardiac-gated breath-hold TSE black blood
Tsim S. et al., 2018 [8]	Prospective cohort study	Not reported	66	60	31	3T MR and CT	T1 weighted, fat saturated, 3D spoiled gradient echo sequences and images acquired after injection of contrast
Knuuttila A. et al., 2001 [39]		January 1997 December 1998	34	34	18	1.5T MR and CT	T1-weighted 2D FLASH images, T2-weighted true FISP and T2-weighted fat-suppressed HASTE sequences in the axial plane; After contrast agent injection T1-weighted fat-suppressed 2D FLASH images
Boraschi P. et al., 1999 [40]		Not reported	30	30	11	0.5T MR for 26 patients 1,5T MR for 4 patients	Conventional spin-echo (SE) technique: cardiac-gated TI-weighted images before and after an intravenous injection of contrast, cardiac-gated proton density and T2-weighted images
Coolen J. et al., 2012 [7]	Prospective study	November 2009–May 2010	31	31	14	3T MR and PET/TC	Pre-contrast T2-weighted single-shot turbo spin-echo and diffusion weighted (DW) sequences spin-echo, followed by a dynamic contrast enhancement (DCE) T1-weighted fast field-echo sequence and postcontrast T1-weighted fast field-echo sequence
Coolen J.et al., 2015 [41]	Prospective study	November 2009–December 2012	109	100	67	3T MR and PET/TC and CT	Nonenhanced T2-weighted single-shot turbo spin-echo (and fat sup pression by means of spectral selection attenuated inversion recovery, or SPAIR) and DW spin-echo echo-planar imaging sequence
Hierholzer J. et al., 2000 [6]	Retrospective study	January 1992–June 1998	88	42	9	1.5T MR and CT	Heart rate-dependent T1-weighted sequence, T2-weighted images with a nongated turbo spin echo sequence, contrast-enhanced T1-weighted imaging
Revelli M. et al., 2016 [9]	Retrospective study	May 2011–January 2016	56	56	44	1.5T MR	Pre-contrast multiplanar fast field echo (FFE) T1-weighted, turbo spin echo single shot T2-weighted and sensitivity encoding balanced turbo field echo two-dimensional sequences, an axial DWI sequence, multiplanar FFE three-dimensional T1-weighted sequences with fat suppression (THRIVE) acquired before and after injection of contrast
Koc M. et al., 2017 [42]	Retrospective study	May 2014 and June 2015	62	62	30	1.5T MR	DWI
Gill R. R. et al., 2010 [43]		June 2008–January 2009	62	62	57	3T MR	T2-weighted single-shot acquisition (HASTE), 3D T1-weighted volume-interpolated gradient-echo acquisitions, DW images were acquired with fat suppression and a free-breathing single shot spin- echo EPI sequence
Mehndiratta A. et al., 2009 [44]	Prospective clinical study	Not reported	19	19	19	1.5T MR	DCE-MRI T1-weighted 2D gradient echo sequence
Giesel F.L. et al., 2006 [45]		Not reported	19	19	19	1.5T MR	DCE-MRI T1-weighted two-dimensional fat gradient-echo sequence
Plathow C. et al., 2008 [46]		Not reported	50	50	50	1.5T MR and CT	HASTE, VIBE before and after contrast media, T2- weighted TSE with respiratory gating
Plathow C. et al., 2008 [47]		Not reported	54	54	54	1.5T MR and PET/TC and TC	HASTE, VIBE before and after contrast media, T2- weighted TSE with respiratory gating
Stewart. D. et al., 2003 [48]		45 months	76	76	76	1.5T MR and CT	T1- weighted breath-hold 2D FLASH before and after intravenous administration of contrast
Heelan R. T. et al., 1999 [16]	Prospective staging protocol	Not reported	95	65	65	1.5T MR and CT	T1-weighted, T2-weighted spin-echo cardiac gated and respiratory compensated
Patel A. M. et al., 2017 [49]	Retrospective study	2000–2016	42	12	12	1.5T or 3T MR	Pre-contrast and post-contrast fat saturated axial T1-weighted gradient echo (GRE)
Ohno Y. et al., 2019 [15]	Comparative study	January 2011–December 2017	23	23	23	3T MR, PET/MR, PET/CT, FDG PET/CT	Dual-phase T1-weighted fast gradient-echo sequence, 3D T1-weighted spoiled gradient-echo, sequentially reordered half-Fourier multi-shot STIRFASE, sequentially reordered half-Fourier single-shot STIR spin-echo EPI, contrast-enhanced 3D with DFS sequence
Usuda K. et al., 2019 [50]		Not reported	43	43	11	1.5T MR, CT, FDG-PET/CT	T1-weighted spin-echo sequence, T2-weighted fast spin-echo sequences, DWI using a single-shot echo-planar technique performed under SPAIR with respiratory triggered scan
Vivoda T. et al., 2019 [49]	Prospective study	October 2013 until July 2015	29	19	19	3T MR	T2-weighted turbo spin echo sequence with fat saturation; T1-weighted 3D gradient-echo breath hold sequence; DCE-MRI T1-weighted 3D gradient echo sequence and 3D gradient-echo breath-hold post-contrast T1-weighted
Falaschi F. et al., 1996 [51]		June 1992–January 1994	45	34	9	0.5T MR and CT	T1-weighted, proton density-weighted, T2-weighted, and enhanced T1-weighted spin-echo
Knuuttila A. et al., 1998 [52]	Comparative study	September 1996–December1997	14		14	1.5T MR and CT	2D Flush, HASTE, true-FISP
Tsim S. et al., 2020 [13]	Prospective observational study	January 2013 and October 2016	58	31	31	3T MR and CT	T1-weighted, fat saturated, 3D-spoiled gradient echo sequences pre and post contrast
